# The resolution of evolutionary conflicts within species

**DOI:** 10.1098/rspb.2024.1594

**Published:** 2024-09-18

**Authors:** J. Arvid Ågren, Göran Arnqvist, Locke Rowe

**Affiliations:** ^1^ Lerner Research Institute, Cleveland Clinic Foundation, Cleveland, OH, USA; ^2^ Evolutionary Biology, Department of Ecology and Genetics, Uppsala University, Uppsala, Sweden; ^3^ Animal Ecology, Department of Ecology and Genetics, Uppsala University, Uppsala, Sweden; ^4^ Department of Ecology and Evolutionary Biology, University of Toronto, Toronto, Ontario, Canada; ^5^ Swedish Collegium of Advanced Study, Uppsala, Sweden

**Keywords:** conflict, multicellularity, development, sex

## Abstract

Evolutionary conflicts of interest occur at all levels, scales and forms of biological organization. They are a fundamental component of the living world and range from conflicts between genetic elements and cells, to conflicts between the sexes and between competing individuals. Yet, the existence of admirably well functioning genomes, bodies, mating pairs and societies suggests that processes must exist to resolve or mitigate such conflicts. We organized this special feature ‘The resolution of evolutionary conflicts within species’ to encourage the flow of knowledge between fields that traditionally have often taken different approaches to study evolutionary conflicts. Contributed papers discuss data from bacteria, plants and animals (including humans) and present theory, molecular mechanisms and population dynamics of how conflicts are resolved in nature. Together, they contribute to a synthetic theory of conflict resolution.

Cooperation is a classic problem for evolution theory. Thanks to this status, the topic has received extensive attention by biologists, with the consequence that we now have a comprehensive understanding of the mechanisms governing its origin and maintenance [1]. Evolutionary conflicts, in contrast, have seen the opposite trajectory. Because selfish behaviour is often taken as the default Darwinian expectation, a synthetic theory of conflict has lagged behind [2].

Evolutionary conflict is rooted in inherent properties of the phenomenon studied. At its most basal level, conflict can be seen as latent and unresolvable as the battleground stems from the basic rules of the game, such as the presence of multicellularity, sexual reproduction, DNA replication, ontogeny, anisogamy, sociality, and so on [3,4]. Within species, conflicts thus occur between individuals over access to mates and resources, between males and females over reproductive decisions, and between parents and offspring over parental investment [5,6]. Within organisms, conflicts occur between different genetic elements (intra- and intergenomic conflict) and cell types, or over the developmental trajectory of specific traits [7]. Exact definitions of evolutionary conflict and its resolution vary across sub-fields of biology, as is illustrated in this special feature. Broadly, however, evolutionary conflicts arise from divergent fitness interests of the interacting parties, and manifestations of potential conflict can lead to a variety of ‘tragedy of the commons’ scenarios [8] that come at a cost to fitness of one or both parties. Such costs can depress population fitness and represent various forms of load [9], or resources lost to conflict [10]. Conflict resolution, then, can be thought of as an evolutionary process that reduces or restricts such costs and enables the functioning of genomes, individuals, mating pairs and societies.

For this special feature titled ‘The resolution of evolutionary conflicts within species’, we opted for a broad definition of conflict, without sharp edges. Instead, we encouraged contributors to articulate how these terms are used in their own domain. We believe that there is much to be learned by collecting and considering the origins of different forms of conflicts side-by-side, comparing the mechanisms that evolved to resolve them, and examining how different fields have conceptualized those processes. Both commonalities and differences should promote new ideas in each area of study. With this in mind, we assembled a diverse set of contributors straddling the full breadth of the field. Across ten papers, they tackle the topic in systems spanning from microbes to plants to humans, reviewing molecular mechanisms and population dynamics, demonstrating just how comprehensive the study of conflict resolution is. The range of mechanisms employed to reduce the costs or resolve conflict include gene duplication [11], sex-biased expression of genes [12,13], differentiation of male and female structures in hermaphrodites [14], context-specific dominance [13,15], suppressors of meiotic drive [16] and transposable elements [17], separation of germline and somatic cells [18], and policing [19], or improved assessment and information [20] in social interactions.

One reason why we need a better understanding of conflict resolution is that the balance between conflict and cooperation is built into the fabric of life. There is a large literature on the so-called major transitions in individuality, events where previously independently living entities came together to form new, higher levels of individuality (including genes in genomes, genomes in eukaryotic cells, cells in multicellular bodies, and organisms in groups and societies) [21,22]. During all transitions, cooperation has in essence overpowered conflict. A major upshot of this commonality is we can use the same tools of social evolution across the resulting hierarchy, from cells to societies. This approach has, for example, demonstrated the importance of relatedness in the evolution of both obligate multicellularity [23] and eusociality in insect societies [24]. Even so, the evolutionary importance of conflict and its resolution have often been downplayed [25]. Both multicellularity and eusociality are what Queller [26] called fraternal (between related parts, like the origin of multicellularity), as opposed to egalitarian (between unrelated parts, like the symbiotic origin of the eukaryotic cell) transitions. It has recently been suggested that the role of conflict resolution is marginal, especially during fraternal transitions [27]. In his contribution, Bourke argues that while this is true in a narrow sense, the conflict-free origins of multicellularity and eusociality were only possible because lower-level conflicts between unrelated parts (both intra- and inter-genomic conflicts) had already been resolved [19]. It is precisely in lineages where lower-level conflicts have been appropriately resolved that cooperation can flourish.

For specific conflicts, several means of conflict resolution have often been identified. This diversity raises the question of the relative importance of different mechanisms. For example, the transition to multicellularity has occurred numerous times and in multiple kingdoms across the tree of life. Only in a limited number of cases, however, has it resulted in complex multicellularity, as measured by cell number and type. Why is that? What are the processes that allow certain lineages to evolve bodies made of billions of cells and thousands of cell types, whereas other bodies are made of only a couple of non-differentiated cells? Howe *et al*. use a large-scale phylogenetic comparative approach to test the role of two conflict-suppressing mechanisms (the presence of a single-cell bottleneck and the separation of germ and soma) in governing this variation in 138 species across Animalia, Bacteria, Chromista, Fungi and Plantae [15]. They find no role of a single-cell bottleneck, but in a pattern driven largely by variation in the Metazoa, early segregation of the germline is associated with greater diversity of cell types. Thus, whereas both have undoubtedly played a role, their importance in evolutionary history is not the same.

Another ancient source of conflict is the evolution of sex [5]. As laid out by Pennell *et al*. [13], the evolution of sex means that a shared genome almost inevitably will be pulled in opposite directions owing to sex-specific divergent selection [13]. What is good for a male is not necessarily what is good for a female. Much progress has been made in teasing out the various ways this form of sexual conflict can be managed, and Pennell *et al*. [13] review the full gamut. Three ways for the cost of trade-offs between the sexes to be reduced are discussed in more detail by Castellanos *et al*. (gene duplications) [11], Cutter (differential gene expression) [12] and Grieshop *et al*. (dominance reversal) [15]. These three contributions focus on different evolutionary conflicts or subsets of those conflicts, including intralocus sexual conflict, ontogenetic conflict and those conflicts that arise from a single genome being tasked to create different phenotypes in different contexts (sex or life history stage). While differing in detail, they all highlight how, from a gene’s point of view, the sex of the body in which it finds itself is as much part of the environment as the temperature or humidity of its surroundings [28]. Consequently, all may involve antagonistic selection at one or several loci, being pulled in one direction in one context and another direction in another context.

Conflicts also vary across space and time. Take hermaphrodites, who express both male and female reproductive functions in a single individual and therefore experience a distinct form of sexual conflict [29]. For example, in hermaphroditic plants the optimal flower phenotype may differ for male and female functions, generating conflicting selection over sex allocation. Chen & Pannell report results from their studies of the plant *Pulsatilla alpina*, which produces both bisexual and male flowers, and demonstrate the presence of such conflicting selection in bisexual flowers [14]. They further analysed the selective effects of flowering time and found that individuals delayed the onset of the male function in bisexual flowers until their pistils (the female reproductive organ) had been exposed to pollinators. While this solves one potential problem (self-fertilization), it generates a new kind of conflict, as the delayed onset results in limited availability of mating partners. Here, Chen & Pannell argue that the resolution of conflict involves producing male-only flowers early in the season, while the bisexual flowers are expressing female function. In spirit, this is analogous to some cases of sex-biased gene expression covered by Cutter [12] or the gene duplication in separate-sexed taxa discussed by Castellanos *et al*. [11] where one paralogue then undergoes neofunctionalization and sex-specific expression in, for example, testes.

Another major arena for conflicts is sex chromosomes. Martí & Larracuente [16] discuss how the lack of recombination and homology between sex chromosomes makes them the perfect place to dissect the molecular biology of conflict. They demonstrate how new genomic data have revealed just how much of evolutionary history has been characterized by cycles of conflicts between sex chromosomes over transmission via the male germline, through the acquisition and amplification of multigene families. What makes this empirical progress especially exciting is that, while the study of meiotic drive has a long experimental history, with a rich theoretical framework to supplement it, new data can still lead to new theoretical advances.

A conflict that has seen a similar data–theory synergistic development is the one caused by the activity of transposable elements. Transposons may be the kind of intragenomic conflict with the strongest theoretical foundation. Comprehensive mathematical population genetic models were developed in the 1980s, and their theoretical predictions have generally held up well in the genomic era [30–32]. Even so, we have learned new things. One example is the importance of epigenetic silencing of transposons. The suppression of transposons in this way minimizes their harmful effects and has certainly been crucial in resolving conflict. However, as Huang & Lee [17] reveal through computer simulations, the selective benefit of silencing is surprisingly multifaceted. Silencing not only suppresses the activity of selfish transposons, but its effects often spill over onto other nearby genes, disrupting their function. The resulting picture is one where, depending on the specific context of transposon copy number and the molecular mechanism of suppression, epigenetic silencing of transposable elements can be both a blessing and a curse.

Genes like organisms, Hurst *et al*. wrote in their classic 1996 review of genetic conflicts, cooperate for one of two reasons: it is in their own interest to, or they are forced to do so [33]. Having examined the various molecular mechanisms that exist to force genes to cooperate, why would it be in their interest to do so? One is the shared bottleneck probed by Howe *et al*. [18], another is full co-transmission, as in the case of asexual genomes (where conflicts are indeed virtually absent). The logic in both cases is that when the fitness of one individual is linked to another, there will be no gain in cheating. In the human cooperation literature, this idea has often been conceptualized as interdependence, a general term that is used to describe the notion that cost or benefit to others may yield a cost or benefit to you in turn. (In this way, it shares much with the evolutionary genetic concepts of kin effects and indirect genetic effects.) So far, the concept has typically been put to work to illustrate the advantages of positive interdependence—benefits for me result in benefits to you. As Stewart *et al*. show in their contribution, negative interdependence (benefits to me results in costs for you, or vice versa) is a potent source of conflict [20]. They demonstrate that when humans have an inaccurate perception of the ‘stake’ they have in someone else’s success (i.e. their interdependence), this can lead to needless conflicts. These results raise an interesting question about the role of information in conflict and cooperation. While typically a good thing, lack of information may sometimes help cooperation by constraining selfishness through a veil of ignorance [34,35].

Taken together, the contributions to this special feature highlight several points of contact between disparate fields that appear worth pursuing further. For example, there is a long history of developmental genetics that has yielded a detailed understanding of how divergent phenotypes manifested across ontogeny can be created by a single genome. Conceptually, this shares many features with the kind of issues encountered by researchers in fields like intralocus sexual conflict, meaning that there is potential for the molecular details of developmental genetics to be married with the theory of sexual conflict. For conflicts resulting from antagonistic selection, in general, there are also broad analogies with spatially or temporally fluctuating selection [36]. The common lesson in all cases is that context matters, which is also echoed in the role of differential gene expression and dominance reversal.

A particular mechanism may also have functions beyond conflict resolution. For example, the epigenetic machinery involved in silencing transposable elements also plays key roles in gene regulation across the genome [37]. Similarly, plasticity is a form of context dependence with a very large empirical and theoretical literature. Again, conflict theory may help make sense of empirical observations that typically are not interpreted with such a lens. Conversely, those interested in the theoretical aspects of molecular conflicts should peruse the gene regulation and plasticity literature for data to incorporate into their models.

Another theme of the special feature is that conflict resolutions often only deal with the manifestation, rather than the source, of evolutionary conflicts. Even with trade-off constraints broken, transposons silenced, or information accurately transmitted, there are still separate sexes, and genes and individuals with different fitness optima. If a gene is duplicated and neo-functionalized, intralocus sexual conflict at the parent locus may be entirely resolved at that locus, but conflict at other loci remains. The same is true for the silencing of a given transposon: there are still many more. It is practically impossible to make the DNA replication machinery failure-proof, or meiosis fully fair, and in the absence of clonality, individuals will always come into conflict on occasion. A rare example is the mechanisms (developmental bottlenecks and the separation of germ and soma) studied by Howe *et al*. [18], which affect the rules of the game in a more direct way. Even so, the clonality of the multicellular body is fragile, and cancer and microchimeric cells constantly challenge its integrity.

From cells to sex to societies, the rules of the game mean that conflict is an inherit part of evolutionary systems. Conflict resolution will, therefore, always be integral to biological function. By bringing together researchers from across the field, we have taken a first step towards a common theory of evolutionary conflicts and their resolution.

## Data Availability

This article has no additional data.

## References

[1] West SA , Cooper GA , Ghoul MB , Griffin AS . 2021 Ten recent insights for our understanding of cooperation. Nat. Ecol. Evol. **5** , 419–430. (10.1038/s41559-020-01384-x) 33510431 PMC7612052

[2] Queller DC , Strassmann JE . 2018 Evolutionary conflict. Annu. Rev. Ecol. Evol. Syst. **49** , 73–93. (10.1146/annurev-ecolsys-110617-062527)

[3] Mock DW , Forbes LS . 1992 Parent-offspring conflict: a case of arrested development. Trends Ecol. Evol. **7** , 409–413. (10.1016/0169-5347(92)90022-4) 21236082

[4] Lessells CM . 1999 Sexual conflict in animals. In Levels of selection in evolution (ed. L Keller ), pp. 73–99. Princeton, NJ: Princeton University Press. (10.1515/9780691207018-007)

[5] Arnqvist G , Rowe L . 2005 Sexual conflict. Princeton, NJ: Princeton University Press.

[6] Brockhurst MA , Chapman T , King KC , Mank JE , Paterson S , Hurst GDD . 2014 Running with the Red Queen: the role of biotic conflicts in evolution. Proc. R. Soc. B **281** , 20141382. (10.1098/rspb.2014.1382) PMC4240979 25355473

[7] Patten MM , Schenkel MA , Ågren JA . 2023 Adaptation in the face of internal conflict: the paradox of the organism revisited. Biol. Rev. **98** , 1796–1811. (10.1111/brv.12983) 37203364

[8] Rankin DJ , Bargum K , Kokko H . 2007 The tragedy of the commons in evolutionary biology. Trends Ecol. Evol. **22** , 643–651. (10.1016/j.tree.2007.07.009) 17981363

[9] Rice WR . 1992 Sexually antagonistic genes: experimental evidence. Science **256** , 1436–1439. (10.1126/science.1604317) 1604317

[10] Ratnieks FLW , Foster KR , Wenseleers T . 2006 Conflict resolution in insect societies. Annu. Rev. Entomol. **51** , 581–608. (10.1146/annurev.ento.51.110104.151003) 16332224

[11] Castellanos MdP , Wickramasinghe CD , Betrán E . 2024 The roles of gene duplications in the dynamics of evolutionary conflicts. Proc. R. Soc. B **291** , 20240555. (10.1098/rspb.2024.0555) PMC11285756 38865605

[12] Cutter AD . 2023 Sexual conflict, heterochrony and tissue specificity as evolutionary problems of adaptive plasticity in development. Proc. R. Soc. B **290** , 20231854. (10.1098/rspb.2023.1854) PMC10565415 37817601

[13] Pennell TM , Mank JE , Alonzo SH , Hosken DJ . 2024 On the resolution of sexual conflict over shared traits. Proc. R. Soc. B **291** , 20240438. (10.1098/rspb.2024.0438) PMC11289733 39082243

[14] Chen KH , Pannell JR . 2023 Unisexual flowers as a resolution to intralocus sexual conflict in hermaphrodites. Proc. R. Soc. B **290** , 20232137. (10.1098/rspb.2023.2137) PMC10685137 38018108

[15] Grieshop K , Ho EKH , Kasimatis KR . 2024 Dominance reversals: the resolution of genetic conflict and maintenance of genetic variation. Proc. R. Soc. B **291** , 20232816. (10.1098/rspb.2023.2816) PMC10932723 38471544

[16] Martí E , Larracuente AM . 2023 Genetic conflict and the origin of multigene families: implications for sex chromosome evolution. Proc. R. Soc. B **290** , 20231823. (10.1098/rspb.2023.1823) PMC10618873 37909083

[17] Huang Y , Lee YCG . 2024 Blessing or curse: how the epigenetic resolution of host-transposable element conflicts shapes their evolutionary dynamics. Proc. R. Soc. B **291** , 20232775. (10.1098/rspb.2023.2775) PMC11003778 38593848

[18] Howe J , Cornwallis CK , Griffin AS . 2024 Conflict-reducing innovations in development enable increased multicellular complexity. Proc. R. Soc. B **291** , 20232466. (10.1098/rspb.2023.2466) PMC10777161 38196363

[19] Bourke AFG . 2023 Conflict and conflict resolution in the major transitions. Proc. R. Soc. B **290** , 20231420. (10.1098/rspb.2023.1420) PMC10565403 37817595

[20] Stewart AJ , Pilgrim C , Raihani NJ . 2024 Resolving selfish and spiteful interdependent conflict. Proc. R. Soc. B **291** , 20240295. (10.1098/rspb.2024.0295) PMC11003781 38593846

[21] Maynard Smith J , Szathmáry E . 1995 The major transitions in evolution. Oxford, UK: Oxford University Press.

[22] Bourke AFG . 2011 Principles of social evolution. Oxford, UK: Oxford University Press.

[23] Fisher RM , Cornwallis CK , West SA . 2013 Group formation, relatedness, and the evolution of multicellularity. Curr. Biol. **23** , 1120–1125. (10.1016/j.cub.2013.05.004) 23746639

[24] Hughes WOH , Oldroyd BP , Beekman M , Ratnieks FLW . 2008 Ancestral monogamy shows kin selection is key to the evolution of eusociality. Science **320** , 1213–1216. (10.1126/science.1156108) 18511689

[25] Ågren JA , Davies NG , Foster KR . 2019 Enforcement is central to the evolution of cooperation. Nat. Ecol. Evol. **3** , 1018–1029. (10.1038/s41559-019-0907-1) 31239554

[26] Queller DC . 1997 Cooperators since life began. Q. Rev. Biol. **72** , 184–188. (10.1086/419766)

[27] Boomsma JJ . 2022 Domains and major transitions of social evolution. Oxford, UK: Oxford University Press. (10.1093/oso/9780198746171.001.0001)

[28] Ågren JA . 2021 The gene’s-eye view of evolution. Oxford, UK: Oxford University Press. (10.1093/oso/9780198862260.001.0001)

[29] Schärer L , Janicke T , Ramm SA . 2014 Sexual conflict in hermaphrodites. Cold Spring Harb. Perspect. Biol. **7** , a017673. (10.1101/cshperspect.a017673) 25237131 PMC4292172

[30] Charlesworth B , Charlesworth D . 1983 The population dynamics of transposable elements. Genet. Res. **42** , 1–27. (10.1017/S0016672300021455)

[31] Lee YCG , Langley CH . 2010 Transposable elements in natural populations of Drosophila melanogaster. Phil. Trans. R. Soc. B **365** , 1219–1228. (10.1098/rstb.2009.0318) 20308097 PMC2871824

[32] Munasinghe M , Springer N , Brandvain Y . 2023 Critical role of insertion preference for invasion trajectory of transposons. Evolution **77** , 2173–2185. (10.1093/evolut/qpad128) 37519088

[33] Hurst LD , Atlan A , Bengtsson BO . 1996 Genetic conflicts. Q. Rev. Biol. **71** , 317–364. (10.1086/419442) 8828237

[34] Queller DC , Strassmann JE . 2013 The veil of ignorance can favour biological cooperation. Biol. Lett. **9** , 20130365. (10.1098/rsbl.2013.0365) 24132090 PMC3871334

[35] Ågren JA , Haig D , McCoy DE . 2022 Meiosis solved the problem of gerrymandering. J. Genet. **101** , 38. (10.1007/s12041-022-01383-w) 36156509

[36] Wittmann MJ , Bergland AO , Feldman MW , Schmidt PS , Petrov DA . 2017 Seasonally fluctuating selection can maintain polymorphism at many loci via segregation lift. Proc. Natl Acad. Sci. USA **114** , E9932–E9941. (10.1073/pnas.1702994114) 29087300 PMC5699028

[37] Branco MR , Chuong EB . 2020 Crossroads between transposons and gene regulation. Phil. Trans. R. Soc. B **375** , 20190330. (10.1098/rstb.2019.0330) 32075561 PMC7061990

